# Highly Efficient Treatment of Oily Sludge by a Novel High-Speed Stirring Method at Room Temperature

**DOI:** 10.3390/ijerph192416817

**Published:** 2022-12-14

**Authors:** Yimin Zhu, Keqing Li, Yin Wang, Jiao Zhao, Xiaojia Tang, Tie Li, Chenming Zhang

**Affiliations:** Collaborative Innovation Center for Vessel Pollution Monitoring and Control, Dalian Maritime University, Dalian 116026, China

**Keywords:** oily sludge, residual oil rate, high-speed stirring, two-stage cleaning

## Abstract

Oily sludge is one of the main hazardous wastes which seriously endangers the ecological environment and human health. In this paper, in order to effectively treat oily sludge, a novel high-speed stirring (HSS) method was proposed to clean oily sludge, and the main parameters affecting the residual oil rate of oily sludge were studied experimentally. Firstly, the cleaning time and stirring speed were optimized in the one-stage HSS cleaning, and then the optimal cleaning time of two-stage HSS cleaning was determined by the response surface method. The results suggested that the oil can be efficiently separated by high-speed stirring at room temperature, and that the two-stage cleaning with a circular-hole outlet (Rotor-C) followed by a vertical hole-outlet (Rotor-V) presented the best effect. The optimal stirring speed was 6000 r/min, and the optimal cleaning times of the two-stage cleaning were 7 min and 8 min, respectively. After cleaning, the residual oil rate of the treated oily sludge was 1.65%, and the removal rate of the petroleum hydrocarbons was 84.3%.

## 1. Introduction

Oily sludge is a pollutant formed when oil is mixed with mud or another medium in which valuable petroleum cannot be recovered directly. Oily sludge is generally composed of oil in water (O/W), water in oil (W/O) and suspended solids in a stable suspended emulsion system. Oily sludge is produced during the process of crude oil production, storage, transportation and refining. Because of its high oil content, high viscosity, difficult dehydration and serious emulsification [1,2], it is harmful to the ecological environment and to human health [3,4,5,6].

Currently, the main methods of oily sludge treatment include incineration, solvent extraction, solidification/stabilization, cleaning, landfill, ultrasonic, biological methods and so on. There are some problems with these treatment methods, such as the high cost, the low treatment efficiency, the secondary pollution and so on [7,8,9,10,11,12,13]. The cleaning method is widely studied for its advantages such as simple operation, high treatment efficiency and low cost. In the process of oily sludge cleaning, chemical heat cleaning is generally used, which utilizes hot water and the lipophilic and hydrophilic properties of the surfactants. During the course of the chemical heat cleaning, the force between the three phases of the oil, water and sludge is changed, and the viscosity of the oily sludge is reduced, so as to realize the separation of the oil from the oily sludge [11]. In the 1980s, the United States, France, Germany and other European countries began to use chemical heat cleaning to treat oily sludge [14]. The temperature of the oily sludge treatment by the cleaning method is generally between 50 °C and 80 °C [15,16,17,18,19]. The cleaning agent added during the oily sludge cleaning is generally a single cleaning agent (such as sodium silicate, etc.), or a compound cleaning agent (such as AO-9 + Na_2_SiO_3_, etc.) [20,21]. Because of the different properties of the oily sludge and the cleaning conditions, the cleaning time is generally 30–120 min [16,22,23,24]. After cleaning the oily sludge using chemical heat cleaning, the residual oil rate of the sludge sample is generally between 2–4% [18,19]. Due to the prolonged cleaning time and the different cleaning methods, such as ultrasound-assisted cleaning, the residual oil rate of the cleaned sludge sample is less than 2% [21]. In general, oily sludge cleaning generally adopts the method of hot washing and cleaning agents, which consumes a lot of energy and organic surfactants, generates oily wastewater and takes a long period of time. Therefore, there are serious problems in terms of the cleaning cost, the cleaning effect and the secondary pollution.

In this research, the oily sludge produced in an oilfield in China is treated by a novel high-speed stirring (HSS) cleaning method at room temperature. Through a single-factor experiment and the response surface method, the stirring speed, the cleaning time and the rotor combination modes are mainly studied in oily sludge cleaning. Compared with chemical heat cleaning methods, the HSS method has the advantages of no heating, low energy consumption, a short cleaning period, a low cost, no wastewater discharge, etc. The novelty of the HSS cleaning method is that it is operated at room temperature, with high efficiency and reduced energy consumption. It provides a new method for oily sludge treatment.

## 2. Materials and Methods

### 2.1. Materials and Instruments

Oily sludge samples were taken from an oilfield in China. The three-phase content of oil, water and solid was 10.50%, 8.22% and 81.28%, respectively, tested by the azeotropic distillation. The cleaning agent is silicate, purchased from a chemical company in Shandong, China. The carbon tetrachloride was purchased from the Tianjin Aoran Fine Chemical Research Institute. The magnesium silicate adsorbent was purchased from the Tianjin Kemeiou Chemical Reagent Co., Ltd. in Tianjin, China.

The experimental instruments are as follows: an infrared oil-measuring instrument OIL480 (Beijing Huaxia Kechuang Instrument Co., Ltd. (Beijing, China)); an HY-5A Gyratory Oscillator (Changzhou Langyue Instrument Manufacturing Co., Ltd. (Changzhou, China)); a KQ-500B Ultrasonic Cleaner (Kunshan Ultrasound Instrument Co., Ltd. (Kunshan, China)); a DK-S26 Electric Heating Constant Temperature Water Bath (Shanghai Jingqi Instrument Co., Ltd. (Shanghai, China)); and an H1850 Desktop High-speed Centrifuge (Hunan Xiangyi Laboratory Instrument Development Co., Ltd. (Hunan, China)).

### 2.2. High-Speed Stirring (HSS) Cleaning Device

**Figure 1:**
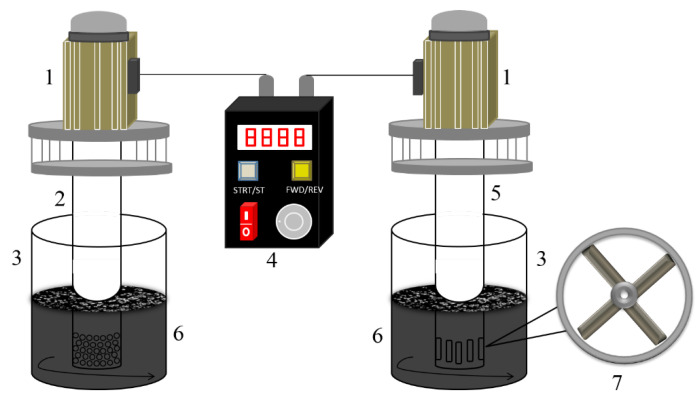
Diagram of a high-speed stirring cleaning device: 1—brushless motor; 2—Rotor-C; 3—mixing tank; 4—motor controller; 5—Rotor-V; 6—oily sludge; 7—impeller.

As shown in Figure 1, the self-made, high-speed stirring device consists of a brushless motor, a motor controller, a stirring rotor and a mixing tank. The stirring rotor consists of a fixed cylinder (with a diameter of 64 mm and a wall thickness of 6 mm) and an impeller (with a diameter of 50 mm). There are four blades in total, each with a thickness of 10 mm. There are two kinds of fixed cylinders with circular-hole and vertical-hole outlets, respectively, on the bottom side, and the corresponding stirring rotor is labeled as Rotor-C and Rotor-V, respectively. The diameter of the circular hole is 3 mm, with 5 rows in a cross arrangement, and 15 holes for each row. The width and the height of the vertical hole is 4 mm and 10 mm, respectively, with 10 holes in a row.

### 2.3. Procedure

In this research, the gravels with particles size larger than 2 mm in the oily sludge were screened out before the experiment. Firstly, the sieved oily sludge (200 g) and the cleaning agent (such as Sodium Metasilicate Pentahydrate, etc.) were added into the mixing tank (with a diameter of 105 mm and a height of 145 mm), according to the mass ratio of 25:3; then, the water was added with the sludge/water ratio of 0.5 g/mL. After premixing, the motor controller was started to carry out the oily sludge cleaning experiment.

The motor drove the stirring rotor to rotate at a high speed in the fixed cylinder. During the cleaning procedure, the oily sludge was sucked from the bottom of the fixed cylinder, and then thrown out at a high speed from the circular-hole or vertical-hole outlet on the fixed cylinder. The oily sludge was circulated and cleaned in the stirring vessel. Then, crude oil floated and skimmed from the liquid surface. The mixture was dewatered by centrifugal separation. Finally, the oil content of the sludge after cleaning was measured.

The content of the mineral oil in the oily sludge was determined by the national standard, according to China’s “Infrared Photometric Method for Determination of Petroleum Oil in Soil (Draft for Comment)” and “National Standard HJ 637-2012. Infrared Photometric Method for Determination of Petroleum and Animal and Vegetable Oil in Water Quality [S]”. For each sample, three parallel experiments were conducted.

## 3. Results and Discussion

### 3.1. Single-Factor Experiment

**Figure 2:**
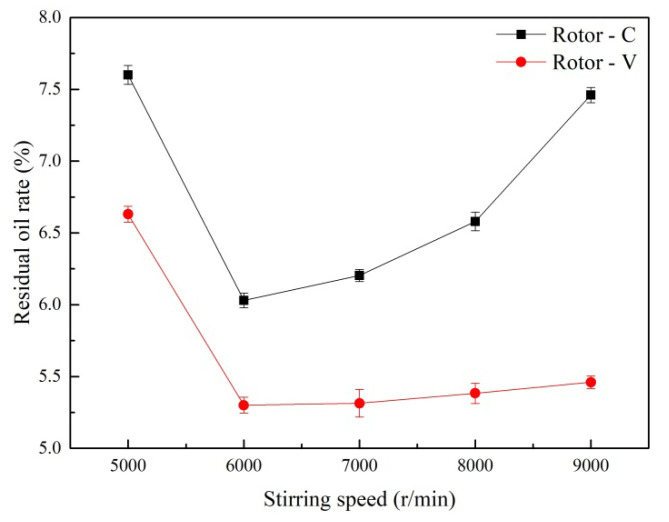
Effects of the stirring speed on the residual oil rate of the treated oily sludge.

The oily sludge samples were treated with the HSS cleaning method, as described in Section 2.3. The cleaning time was set as 5 min, and the experiment was carried out at various stirring speeds. Figure 2 shows the effects of the stirring speed on the residual oil rate of the oily sludge. When the stirring speed increased from 5000 r/min to 9000 r/min, the residual oil rate of the treated oily sludge first decreased, and then increased for both of Rotor-C and Rotor-V. The minimum residual oil rate of the oily sludge cleaned by Rotor-V was 5.30% at the optimum stirring speed of 6000 r/min, which was lower than that cleaned by Rotor-C (6.03%).

In the HSS cleaning process, due to the impeller rotating at a high speed, a low-pressure zone is created at the bottom of the impeller. As a result, the slurry is drawn in from the bottom of the impeller due to pressure changes. At the same time, the slurry is thrown out from the holes of Rotor-C or Rotor-V. Meanwhile, the high-speed rotation of the rotor produces a strong shear force in the slurry, so that the slurry is cleaned circularly and produces centrifugal movement. The slurry is sheared and demulsified in the high-speed stirring, while the oil phase is desorbed from the mud phase.

With the increase in the rotor speed, the shear force is proportionally enhanced [25], which leads to oil desorption and promotes air flotation. When the rotor speed reached 6000 r/min, the residual oil rate of the treated oily sludge was minimized. When the rotor speed further increased, the enhanced shear force continued to emulsify the separated oil and recombined some of the separated oil with the slurry, resulting in a gradual increase in the residual oil rate of the treated oily sludge.

**Figure 3:**
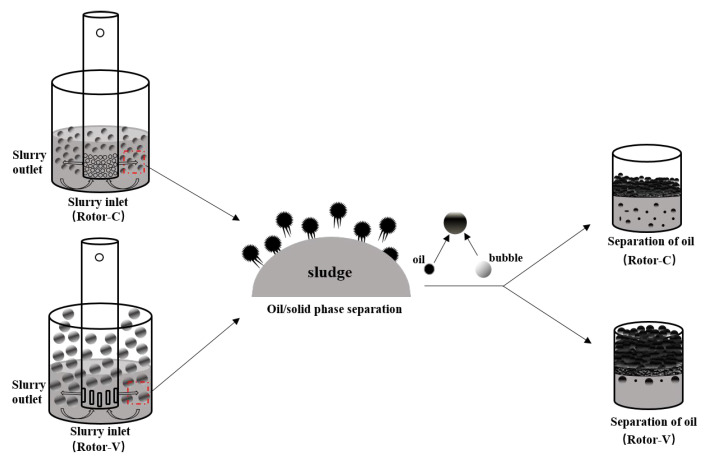
Schematic diagram of the oily sludge cleaning.

In the HSS cleaning process, cavitation occurs due to a transient change in pressure [26]. The cavitation bubble has the function of air floating, which can float and converge the desorbed oil phase to the slurry surface to separate the oil phase from the mud phase. As displayed in Figure 3, because of small bubbles produced by Rotor-C, its buoyancy is small, so the air floating effect is weak. Meanwhile, because of the strong emulsifying effect at a higher speed of Rotor-C, the sludge residual oil rate increased significantly. Because of the large bubbles and the strong air flotation produced by Rotor-V, the residual oil rate of the oily sludge only increased slightly, although it has an emulsifying effect when the rotor rotates at a higher speed.

**Figure 4:**
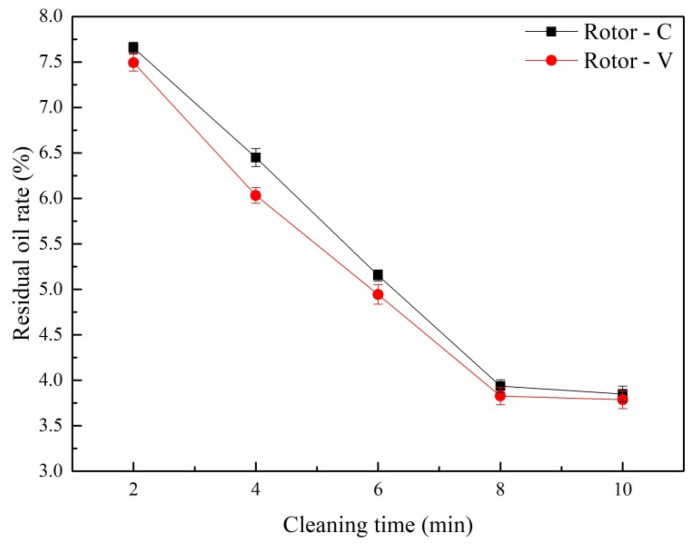
Effects of the cleaning time on the residual oil rate of the oily sludge.

The cleaning time was determined at the stirring speed of 6000 r/min, and the results are provided in Figure 4. When the cleaning time increased from 2 min to 8 min, the residual oil rate of the treated oily sludge decreased linearly, under the combined action of the shearing demulsification and the air flotation separation for both Rotor-C and Rotor-V. However, when the cleaning time exceeded 8 min, the HSS cleaning could not further desorb the residual oil in the oily sludge, so the residual oil rate no longer decreased significantly. Considering the treatment efficiency and the cost, the optimal cleaning time was 8 min. Under the optimum cleaning time, the minimum residual oil rate of the oily sludge was 3.94% and 3.83%, respectively, for Rotor-C and Rotor-V.

### 3.2. Effects of Rotor Combination Mode on Residual Oil Rate of Oily Sludge

Based on the single-factor experiment, the stirring speed and cleaning time were determined for Rotor-C and Rotor-V. In this section, the two-stage combination mode was used to further decrease the residual oil rate of the oily sludge. There were two kinds of rotor combination modes: Rotor-C followed by Rotor-V, and Rotor-V followed by Rotor-C. Table 1 shows the test results of the residual oil rate of the two-stage oily sludge cleaning. After the HSS cleaning by Rotor-V and then Rotor-C, the residual oil rate of the oily sludge was 3.54%, with the petroleum hydrocarbon removal rate of 66.3%. Rotor-C and then Rotor-V mode were beneficial to the oil sludge cleaning, with the residual oil rate of 1.26%, and the petroleum hydrocarbon removal rate of 88.0%.

**Figure 5:**
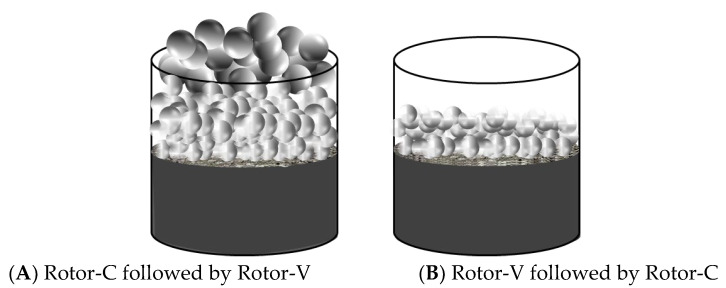
The phenomenon of two-stage HSS for oily sludge cleaning. ((**A**) Rotor-C followed by Rotor-V; (**B**) Rotor-V followed by Rotor-C).

Compared with Rotor-V, the hole diameter of Rotor-C is smaller, so the cavitation phenomenon of Rotor-C in the stirring process is more intense [27]. In the HSS cleaning of the oily sludge, when Rotor-C cleans the oily sludge, the sludge, water and cleaning agent are fully homogeneous, and the oil phase is desorbed from the sludge phase. However, due to the small volume of the bubbles generated by Rotor-C, the bubbles have little buoyancy, which is not conducive to the oil-phase flotation to the surface of the oily sludge. As shown in Figure 5, the hole diameter of Rotor-V is larger, so the cavitation bubbles generated are also larger [27]. With the bubbles floating up, the separated oil phase floats easily to the slurry surface and separates from the slurry. Therefore, when Rotor-C followed by Rotor-V is used for cleaning, the cleaning effect of the oily sludge is the best.

### 3.3. Response Surface Method to Determine the Optimal Cleaning Time of the Combined Rotor

As found in the two-stage oily sludge cleaning research, the best combination of the rotors for the HSS cleaning oily sludge was to use Rotor-C followed by Rotor-V. Under the fixed experimental conditions of the optimum stirring speed of 6000 r/min, the response surface experiment was carried out using the Design-Expert 8.0 software. The cleaning time of Rotor-C and Rotor-V was selected as an important parameter, so as to determine the optimum cleaning time for the rotor combination.

The central composite design (CCD) approach in the Design-Expert 8.0 software was used to establish the relationship between the independent variables and the residual oil rate, and each independent variable was arranged into five levels (−α, −1, 0, 1 and α), as indicated in Table 2. As a result, an experimental matrix including four cubic points, four axial points and five center points was created. The central point was repeated five times during the experiment. The HSS cleaning experiments were then carried out in accordance with the experimental matrix, with the results displayed in Table 3.

Based on the experimental results in Table 3, a quadratic model was established, and ANOVA was applied to assess this response surface model (Table 4).

The response surface model’s F value and *p* value (Prob > F) are 4.81 and 0.0316, respectively, showing that the model has high reliability and significance, and can successfully describe the relationship between the independent variable and the dependent variable. The corresponding *p* value for the Lack of Fit item is 0.4878 (>0.05), suggesting that the difference is not significant, and that the quadratic multinomial regression model well fits the actual data.

**Figure 6:**
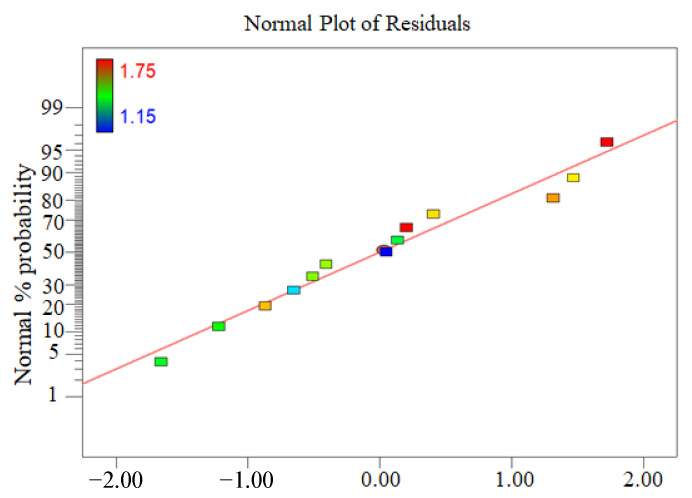
Normal probability distribution of the residual oil rate.

**Figure 7:**
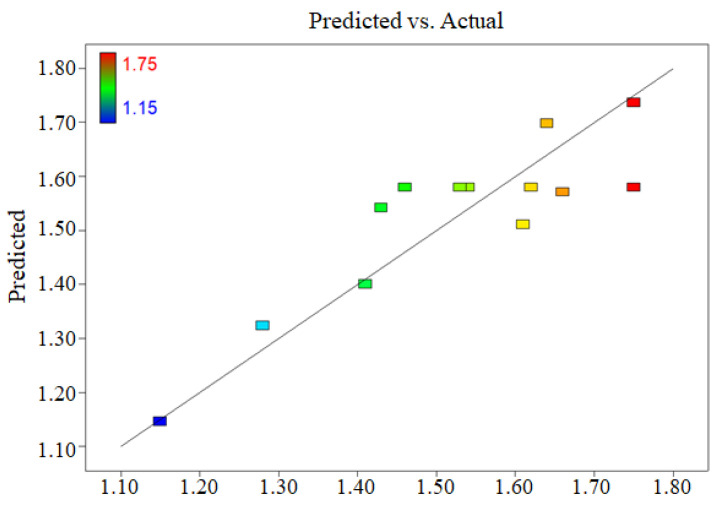
Actual and predicted residual oil rates.

The normal probability distribution diagram of the residual oil rate showed that the normal distribution of the data is almost a straight line (Figure 6), indicating that the model does not require response modification and that there are no evident difficulties with normality. Figure 7 depicted the relationship between the actual and the predicted residual oil rate. The data are nearly linear, implying that the anticipated value is more accurate.

According to the regression analysis of the experimental data, the mathematical model for predicting the residual oil rate was obtained as Equations (1) and (2):

Final equation in terms of the coded factors:

R = 1.58 − 0.13 × *A +* 0.058 × *B* + 0.020 × *AB* − 0.13 × *A^2^* + 0.037*B^2^* (1)

Final equation in terms of the actual factors:

R = 1.523 + 0.282 × *A* − 0.170 × *B* + 5.000 × 10^−3^ × *AB* − 0.031 × *A^2^* + 9.219 × 10^−3^*B^2^* (2)

where, R is the residual oil rate; *A* is the cleaning time of Rotor-V; and *B* is the cleaning time of Rotor-C.

**Figure 8:**
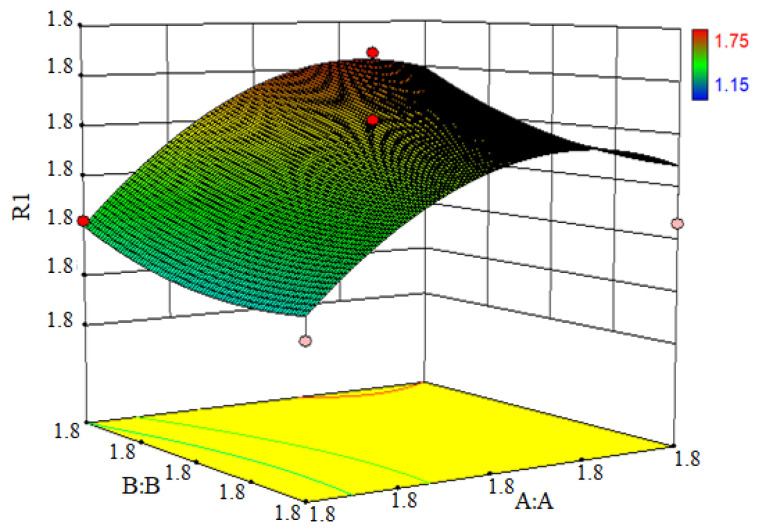
A 3D map of the response surface.

The Design-Expert response surface approach was utilized to explore the impact of the individual factors and the interactions on the residual oil rate of the oily sludge. The 3D response surface diagram (Figure 8) depicted the interaction of the rotor cleaning time with Rotor-V and Rotor-C on the residual oil rate of oily sludge.

When the cleaning time of Rotor-C was fixed, the residual oil of the oily sludge first increased, and then decreased as the cleaning time of Rotor-V increased. The residual oil of the oily sludge fell initially when the cleaning time of Rotor-V was fixed, and then increased when the cleaning time of Rotor-C was prolonged. To summarize, the cleaning time of Rotor-V and Rotor-C had a significant interaction impact on the residual oil rate of the oily sludge.

According to the results of the model optimization, the best cleaning conditions in the range of −1 to 1 were 7 min for Rotor-C and 8 min for Rotor-V. The model predicted a residual oil rate of 1.32%, whereas the actual result was 1.65%. The standard deviation between the observed and the predicted values was 0.23, indicating that the model was reliable.

## 4. Conclusions

This paper used the HSS method to clean oily sludge at room temperature. The optimum stirring speed, cleaning time and rotor combination mode of the oily sludge cleaning were determined by a single-factor experiment. Meanwhile, the response surface method was used to determine the optimum cleaning time of the two-stage oily sludge cleaning. The main conclusions are as follows: (1) The optimum speed of Rotor-C or Rotor-V for cleaning oily sludge is 6000 r/min, and the optimum cleaning time is 8 min. (2) In the two-stage cleaning of oily sludge, Rotor-C, followed by Rotor-V, has the best cleaning effect. The optimum cleaning time for the first stage is 7 min, and for the second stage, it is 8 min, with a stirring speed of 6000 r/min. Under the optimum conditions, the residual oil rate of the oily sludge is 1.65%. (3) According to the response surface experiment, the cleaning time of Rotor-V is longer than that of Rotor-C, and properly extending the cleaning time of Rotor-V helps to improve the cleaning effect. This indicates that Rotor-V mainly plays the role of air floating due to the large bubbles generated in the second-stage cleaning process, which is conducive to the separation of the oil phase. Compared with the chemical heat cleaning methods, the HSS cleaning method can effectively separate oil from oily sludge at room temperature, which has the advantages of no heating, low energy consumption, a short cleaning period, a low cost, no wastewater discharge and good economic benefits and application prospects.

## Figures and Tables

**Table 1:** Test results of the residual oil rate of the two-stage oily sludge cleaning.

Experiment Number	Combination	Stirring Speed (r/min)	Residual Oil Rate%	RSD (%)	Average Residual Oil Rate (%)	Petroleum Hydrocarbon Removal Rate (%)
1	Rotor-C and then Rotor-V	6000	1.26	4.360	1.26	88.0
			1.32			
			1.21			
2	Rotor-V and then Rotor-C	6000	3.57	2.241	3.54	66.3
			3.45			
			3.60			

**Table 2:** Experimental range and the level of the independent variables.

Variables	Factors	Range and Level				
		−1.414	−1	0	1	1.414
Rotor-V cleaning time	A	3.172	4	6	8	8.828
Rotor-C cleaning time	B	3.172	4	6	8	8.828

**Table 3:** Experimental design and results.

Run Number	Rotor-V Cleaning Time (min)	Rotor-C Cleaning Time (min)	Residual Oil Rate (%)	
			Actual	Predicted
1	0	1.414	1.66	1.57
2	0	−1.414	1.75	1.58
3	−1	1	1.43	1.54
4	−1	−1	1.64	1.70
5	0	0	1.53	1.58
6	−1.414	0	1.61	1.51
7	0	0	1.75	1.74
8	0	0	1.62	1.58
9	1	−1	1.41	1.40
10	1	1	1.28	1.32
11	1.414	0	1.15	1.15
12	0	0	1.54	1.58
13	0	0	1.46	1.58

**Table 4:** ANOVA for the response surface quadratic model.

Source	Sum of Squares	df	Mean Square	F Value	*p*-Value Prob > F	
Model	0.29	5	0.058	4.81	0.0316	significant
*A*-*A*	0.13	1	0.13	10.96	0.0129	
*B*-*B*	0.027	1	0.027	2.25	0.1771	
*AB*	1.600 × 10^−3^	1	1.600 × 10^−3^	0.13	0.7270	
*A* ^2^	0.11	1	0.11	9.06	0.0197	
*B* ^2^	9.459 × 10^−3^	1	9.459 × 10^−3^	0.78	0.4063	
Residual	0.085	7	0.012			
Lack of Fit	0.036	3	0.012	0.97	0.4878	not significant
Pure Error	0.049	4	0.012			
Cor Total	0.38	12				

## Data Availability

The data presented in this study are available on request from the corresponding author.
